# Kathryn Hyer's Lasting Legacy in Gerontology Education and Aging Policy Research

**DOI:** 10.1093/geroni/igab046.546

**Published:** 2021-12-17

**Authors:** Debra Dobbs

**Affiliations:** University of South Florida, Tampa, Florida, United States

## Abstract

Kathy Hyer, our dear friend, colleague, former Gerontological Society of America President and Professor and Director of the Florida Policy Exchange Center on Aging, University of South Florida posthumously has been awarded the Clark Tibbett's Award for her achievements in the advancement of the field of gerontological education in higher education. This lecture will reflect on some of her decades of accomplishments including her dedication to AGHE's mission to train and educate students in gerontology. Her greatest achievement is in the area of training and education of long-term care administration and aging studies undergraduate students to be nursing home and assisted living administrators. This lecture will also highlight several initiatives of Dr. Hyer's that involved undergraduate, graduate students, and junior faculty who she mentored including the Geriatric Workforce Enhancement Project, NIA SAFEHAVEN and COVID research on disasters, the Dementia Training Academy, Age Friendly Initiatives both nationally and internationally and student mentorship for graduate assistantships through the USF Center for End of Life Care.

